# Supplementary material for: Resolving the full spectrum of human genome variation using Linked-Reads
Source: Genome Res. 2019 Apr;29(4):635–45. doi: 10.1101/gr.234443.118 (PMC6442396; doi:10.1101/gr.234443.118)
Supplement: Supplemental Material [file supp_gr.234443.118_Supplemental_longranger-2.2.zip › longranger-2.2.tar.bz2!/lr2.2-public/lib/go/src/code.google.com/vitess/web/vtctld/keyspaces.html]

### Loading...

### No keyspaces found.

## {{keyspace.name}}


Keyspace Actions
more\_vert


{{actions.label(action)}}


### Serving Shards

### {{shard}}


### Inactive Shards

### {{shard}}

### No Shards Found


### Shards

### {{shard}}

### No Shards Found
